# Association of interleukin 18 gene promoter polymorphisms with HBV recurrence after liver transplantation in Han Chinese population

**Published:** 2011-06-01

**Authors:** Liming Wu, Lin Chen, Lin Zhou, Haiyang Xie, Bajin Wei, Shengyong Yin, Yufu Ye, Weijia Fang, Shusen Zheng

**Affiliations:** 1 Key Laboratory of Combined Multi-Organ Transplantation, Ministry of Public Health, Hangzhou, China; 2 Department of Hepatobiliary and Pancreatic Surgery, First Affiliated Hospital, School of Medicine, Zhejiang University, Hangzhou, China; 3 School of Medicine, Zhejiang University, Hangzhou, China; 4 Department of Medical Oncology, First Affiliated Hospital, School of Medicine, Zhejiang University, Hangzhou, China

**Keywords:** Interleukin-18, Genetic polymorphism, Hepatitis, Recurrence, Transplantation

## Abstract

**Background:**

There is growing evidence suggesting that interleukin-18 (IL-18) plays a crucial role in viral clearance and disease pathogenesis, and that single nucleotide polymorphisms (SNPs) within the gene may influence its production.

**Objectives:**

To investigate the potential association of two polymorphisms ( 137G/C and 607C/A) in the promoter region of the IL-18 gene with the risk of HBV recurrence after liver transplantation (LT) in Han Chinese patients.

**Patients and Methods:**

IL-18 promoter genotyping was performed by the snapshot technique in 125 patients with HBV-related end-stage liver disease (ESLD) receiving LT in our center from 2004 to 2008.

**Results:**

Among the study samples, no significant association between the IL-18 promoter polymorphisms ( 137G/C and 607C/A) or haplotypes and HBV recurrence after LT was found.

**Conclusions:**

The polymorphisms ( 137G/C and 607C/A) in the promoter region of the IL-18 gene may not play a key role in HBV recurrence after LT in Han Chinese population, and may not be suitable predictors for HBV recurrence in clinical practice.

## 1. Background

Hepatitis B virus (HBV) infection is a leading cause of morbidity and mortality, affecting approximately 350 million people worldwide [1]. Although the majority of patients recover from acute infection with HBV, a significant proportion has an increased risk of developing liver cirrhosis or hepatocellular carcinoma. Currently, liver transplantation (LT) is regarded as the most effective therapy for patients with HBV-related end-stage liver disease (ESLD), which has become the leading indication of LT in China [2]. However, recurrent HBV infection is a major problem in HBV-related ESLD patients undergoing orthotopic liver transplantation. Despite the prophylactic use of hepatitis B immune globulin (HBIG) and nucleoside analogous lamivudine or adefovir, the incidence of post-transplant HBV recurrence varies between 5% and 10% in most centers. The HBV reinfection commonly leads to the rapid development of progressive liver disease resulting in a significant decrease in graft and patient survival. Improved understanding of factors that influence HBV recurrence after LT may help to develop prophylactic and therapeutic approaches to this vexing clinical problem. Furthermore, there is an urgent need to develop biomarkers that may help physicians predict which patients are at high risk for HBV recurrence after LT, enabling them to make earlier treatment.

Despite extensive clinical and basic research efforts, mechanisms of HBV recurrence after LT are still not clear. Factors that influence the HBV reinfection after LT include recipient factors (genetic factors, preoperative HBV replication status, extrahepatic HBV existence status), donor factors (compromised donor liver; HLA-A, -B compatibilities), perioperative treatment (use of antiviral agents, drug resistance, virus mutation, immunosuppressants protocol, blood transfusion), etc., as discussed in our previous review [3]. Over the past few years, both our group and others have focused on searching for reliable genetic biomarkers to better distinguish subtypes of patients with HBV-related ESLD who have different risk of HBV recurrence after LT. Investigators in our group have established a retrospective cohort of HBV-related ESLD patients who underwent LT at our institution, and analyzed some potential biomarkers within this valuable clinical research database. Up until now, genetic SNPs such as CTLA-4 [4], TLR4 [5], and FCGR3A [6] have been implicated in risk of HBV recurrence after LT and employed as a potential tool to guide specific medical therapy after LT in patients with HBV-related ESLD [7].

IL-18, a pro-inflammatory member of the IL-1 cytokine superfamily, is recognized as a key mediator of innate and acquired immune responses [8]. The cytokine IL-18 is broadly expressed on dendritic cells, Kupffer cells, macrophages, endothelial cells, and vascular smooth muscle cells, and is highly upregulated during inflammatory diseases [9]. IL-18 has been implicated in hepatitis virus clearance or disease pathogenesis. McGuinness et al. reported that IL-18 levels were increased in patients with viral hepatitis and correlated with the extent of liver injury [10]. In addition, an in vivo study demonstrated that IL-18 could inhibit HBV replication in the livers of transgenic mice [11]. More recently, it has been reported that polymorphisms at positions 607 and 137 of IL-18 gene are associated with the IL-18 promoter transcription activity, IL-18 expression, and disease progression including rheumatoid arthritis, type 1 diabetes, human immunodeficiency virus (HIV) infection, HCV and HBV clearance [12][13][14][15][16]. However, the relationship between these polymorphisms and risk of HBV recurrence after LT has not been reported.

## 2. Objectives

The aim of the present study was to investigate whether the IL-18 promoter polymorphisms ( 137G/C and 607C/A) is associated with HBV recurrence after LT in Chinese LT patients, and whether it would be a reference in predicting HBV recurrence after LT.

## 3. Patients and Methods

### 3.1. Sample collection

A total of 125 patients with HBV-related ESLD (HBV-related fulminant hepatitis, decompensate liver cirrhosis or hepatocellular carcinoma) who received LT in our center from 2004 to 2008 were enrolled in this retrospective study according to the same eligibility criteria mentioned earlier [5]. All patients were Han Chinese. Informed consent was obtained from all participants; the study was approved by the Ethical Review Committee of the First Affiliated Hospital, School of Medicine, Zhejiang University. The prophylaxis protocol, immunosuppression regimens and diagnosis criteria of HBV recurrence after LT have been shown in our previous study [5]. Patients positive for HBV-DNA would be treated with lamivudine before LT and with HBIG combined with lamivudine (changed into adefovir if YMDD mutation was detected) for the prophylaxis of the recurrence of hepatitis B after LT. Patients with detectable serum HBV DNA at the time of operation were administered 8000 IU of HBIG during the anhepatic phase, in contrast to 6000 IU in those whose serology were negative for HBV DNA before transplantation. A subsequent 800 IU of intramuscular HBIG was administered daily for the first seven post-operative days, twice a week for the next three weeks and then 800 IU monthly thereafter to maintain serum antibody to hepatitis B surface antigen (anti-HBs) concentrations. Maintenance immunosuppression regimens consisted of triple drugs of cyclosporine (CsA), rapamycin or tacrolimus, mycophenolate (MMF) and prednisone. The criteria of HBV recurrence after LT included positive hepatitis B surface antigen (HBsAg) or HBV-DNA ≥105 copies/mL on two occasions. The incidence of HBV recurrence after LT was 9.6% with a mean recurrence time of 7.5 months by the criteria among our study population. A database was built for collecting the clinical data of patients, including their sex, age, blood type, MELD grade, indication for liver transplantation, pre-transplantation HBeAg, types of immunosuppressive agents used and the HBV-related information.

### 3.2. Polymorphism genotyping

Genomic DNA was isolated from EDTA-anticoagulated whole blood using the Maxwell® 16 (Promega Co, USA). The primers were designed using Primer 3 software (http://frodo.wi.mit.edu/primer3/), as shown in Table 1. SNPs at position 607C/A (rs1946518) and 137G/C (rs187238) in the promoter region of the IL-18 gene were genotyped by the technique of snapshot containing two steps (polymerase chain reaction and polymerase chain reaction), as described previously [5].

**Table 1:** Primers for polymerase chain reaction and sequencing of -137G/C and -607C/A in IL-18

	**PCR primers**	**Sequencing primers**
**-137G/C**	Forward: 5’CCCCTTCCTCCCAAGCTCAATA 3’ Reverse: 5’AGGTCAGTCTTTGCTATCATTCCAG 3’	5’TTTTTTTTTTTTTTTTTTTTTTTCCCC-AACTTTTACGGAAGAAAA 3’
	
**-607C/A **	Forward: 5’CAGGTTTTGGAAGGCACAGAGC 3’ Reverse: 5’AGGCTCTTTCCTAGGGCAATGG 3’	5’TTTTTTTTTTTTTTTTTTTTTTCTG-TTGCAGAAAGTGTAAAAATTATTA 3’

### 3.3. Statistical analysis

Statistical analyses were conducted using SPSS 16.0 for Windows® (SPSS, Chicago, IL, USA). The clinical characteristics, genotypes, and frequencies of alleles in patients with and without HBV recurrence were compared using x² test. The Hardy-Weinberg equilibrium, linkage disequilibrium and haplotype were analyzed using Hapoview software and the website for SNPStats (http://bioinfo.iconcologia.net/snpstats/start.htm). A p < 0.05 was considered statistically significant.

## 4. Results

### 4.1. Patient characteristics

A total of 125 patients were enrolled in the study including 111 men and 14 women aged between 23 and 68 years. The primary liver diseases in the present study were HBV-related decompansated liver cirrhosis (60.0%), HBV-related hepatocellular carcinoma (32.8%) and fulminant hepatitis B (7.2%). To exclude the possible influence of the demographic factors, the potential association of 137G/C and 607C/A genotypes with clinical characteristics of patients was analyzed. There were no significant differences in gender, age, blood type, MELD grade, HBeAg status, the use of immunosuppressive agents and primary liver diseases among patients with different genotypes in these two SNP loci (data not shown). Particularly, previous studies have indicated that immunosuppressive therapy leads to high HBV replication [17]. Therefore, in this study, we compared the incidence of HBV recurrence after LT in three groups divided according to the regimen for the maintenance of immunosuppression: CsA/prednisone/MMF group, tacrolimus/prednisone/MMF group, and rapamycin/prednisone/MMF group. However, no significant differences in the incidence of HBV recurrence rate after LT were observed among the three studied groups (data not shown).

### 4.2. IL-18 promoter polymorphisms ( 137G/C and 607C/A) and HBV recurrence

The incidence of HBV recurrence after LT was 9.6% (12/125) with a mean recurrence time of 7.5 months by the criteria for the diagnosis of HBV recurrence. Four patients were excluded from the study because the two SNPs were not detected. The ratio of detection was 96.8% (242/250). Allele frequencies were in Hardy-Weinberg equilibrium in our study sample. The distribution of genotype frequency and allelic frequency between patients with HBV recurrence and nonrecurrence was compared. In the loci of 137G/C, the incidence of HBV recurrence in recipients with C/G and G/G genotypes was 4% (1/23) and 12% (11/96) respectively, and no patients with C/C suffered HBV recurrence. However, no statistical difference was found in either genotype or allelic frequency between patients with HBV recurrence and nonrecurrence. In the loci of 607C/A, the incidence of HBV recurrence in patients with C/C, C/A and A/A genotypes was 7.5% (3/40), 14.8% (8/54) and 3.7% (1/27), respectively. There was also no significant association in either genotype or allelic frequency between recipients with HBV recurrence and nonrecurrence (Table 2).

**Table 2:** The distribution of genotypes and the allelic frequencies in the -137G/C and -607C/A of IL-18 in HBV recurrence patients and non recurrence control subjects

	**Genotype**	**HBV-recurrence **[No. (%)]				**HBV-recurrence **[No. (%)]		
		**No **	**Yes**	**P value**	**Allele**	**No **	**Yes**	**P value **
**-137G/C**				0.529				0.252
	C-C	2 (1.8%)	0 (0%)		C	26 (12%)	1 (4.2%)	
	C-G	22 (20.2%)	1 (8.3%)		_	_	_	
	G-G	85 (80%)	11 (91.7%)		G	192 (88%)	23 (95.8%)	
**-607C/A**				0.237				0.758
	C-C	37 (34%)	3 (25%)		C	120 (55%)	14 (58.3%)	
	C-A	46 (42.2%)	8 (66.7%)		_	_	_	
	A-A	26 (23.8%)	1 (8.3%)		T	98 (45%)	10 (41.7%)	

Different models of inheritance were analyzed to detect the influence of IL-18 polymorphisms ( 137G/C and 607C/A) on HBV recurrence after LT. However, none of them was associated with HBV recurrence after LT (Table 3). Linkage disequilibrium (LD) and haplotype analysis was performed using Haploview program. The 137G/C and 607C/A polymorphisms in the promoter of IL-18 gene were poorly linked (r² = 0.04). Haplotypes were also reconstructed with frequencies more than 0.1 between 137G/C and 607C/A. However, as shown in Table 4, no significant association was found between neither global test nor individual haplotypes and the susceptibility to HBV recurrence after LT.

**Table 3:** The models inheritance for -137G/C and -607C/A in HBV recurrence patients and nonrecurrence control subjects

	**No**	**Yes**	**OR a**(95% CI b)	**P value**	**AIC c**	**BIC d**
**-137G/C Model**						
**Codominant**				0.44	82.6	91
**G/G**	85 (78%)	11 (91.7%)	1.00			
**G/C**	22 (20.2%)	1 (8.3%)	0.35 (0.04–2.87)			
**C/C**	2 (1.8%)	0 (0%)	0.00 (0.00–NA)			
**Dominant**				0.22	80.7	86.3
**G/G**	85 (78%)	11 (91.7%)	1.00			
**G/C-C/C**	24 (22%)	1 (8.3%)	0.32 (0.04–2.62)			
**Recessive**				0.52	81.8	87.4
**G/G-G/C**	107 (98.2%)	12 (100%)	1.00			
**C/C**	2 (1.8%)	0 (0%)	0.00 (0.00–NA)			
**Overdominant**				0.28	81.1	86.7
**G/G-C/C**	87 (79.8%)	11 (91.7%)	1.00			
**G/C**	22 (20.2%)	1 (8.3%)	0.36 (0.04–2.93)			
**Log-additive**	—	—	0.33 (0.04–2.52)	0.21	80.6	86.2
**-607C/A Model**						
**Overdominant**				0.22	81.2	89.6
**C/C**	37 (33.9%)	3 (25%)	1.00			
**C/A**	46 (42.2%)	8 (66.7%)	2.14 (0.53–8.66)			
**A/A**	26 (23.9%)	1 (8.3%)	0.47 (0.05–4.82)			
**Dominant**				0.52	81.8	87.4
**C/C**	37 (33.9%)	3 (25%)	1.00			
**C/A-A/A**	72 (66.1%)	9 (75%)	1.54 (0.39–6.04)			
**Recessive**				0.18	80.4	86
**C/C-C/A**	83 (76.2%)	11 (91.7%)	1.00			
**A/A**	26 (23.9%)	1 (8.3%)	0.29 (0.04–2.36)			
**Overdominant**				0.11	79.6	85.2
**C/C-A/A**	63 (57.8%)	4 (33.3%)	1.00			
**C/A**	46 (42.2%)	8 (66.7%)	2.74 (0.78–9.65)			
**Log-additive**	—	—	0.88 (0.39–2.00)	0.77	82.1	87.7

^a^ Odds ratio

^b^ Confidence Interval

^c^ Akaike’s information criterion

^d^ Bayesian information criterion

**Table 4:** The association between haplotype and HBV recurrence after liver transplantation

**Haplotype a**	**rs187238**	**rs1946518**	**frequency**	**OR b** (95% CI c)	**P value**
**1**	G	C	0.5233	1	—
**2**	G	A	0.365	1.10 (0.48–2.53)	0.82
**Rare**	-	-	0.1117	0.34 (0.04–2.71)	0.31

^a^ Global haplotype association P value: 0.44

^b^ Odds ratio

^c^ Confidence intervals

## 5. Discussion

Our recent efforts have focused on identification of genetic risk factors of recipients in an attempt to improve the power of predicting HBV recurrence after LT in patients with HBV-related ESLD. In the present study, we examined for the first time two polymorphisms ( 137G/C and 607C/A) in the promoter region of the IL-18 gene in the susceptibility to HBV recurrence after LT in Han Chinese patients. No significant association between the IL-18 promoter polymorphisms ( 137G/C and 607C/A) or haplotypes and HBV recurrence after LT was found. Our previous studies supported that cytokine gene polymorphisms may serve as candidate predictive factors for the risk of HBV recurrence after LT [18]. A number of functional polymorphisms within the proximal promoter of the IL-18 gene that may influence IL-18 activity and production have been identified. Among these SNPs, two of the IL-18 promoter at positions 137 and 607 were most studied. Cloning and gene expression analysis demonstrated that variation at 607C/A and 137G/C of IL-18 gene disrupts cAMP-responsive element-binding protein binding site and H4TF-1 nuclear factor binding site, respectively [19]. Human monocytes isolated from 607A carriers produce more IL-18 than 607C carriers. Monocytes from 137G carriers produce more IL-18 than 137C carriers [20]. There is evidence that low production of IL-18 contributes to the viral clearance. IL-18 deficiency is associated with accelerated viral clearance and enhanced activation of CD4+ T cells in mice with influenza A virus infection [11]. Moreover, IL-18 has also been shown to play a role in organ transplantation [21]. However, evidence on the role of IL-18 in liver transplantation is scarce, especially with regard to HBV recurrence after LT. Considering these findings, we hypothesized that the 137G/C and 607C/A polymorphisms in the promoter of IL-18 gene might be associated with the risk of HBV recurrence after LT in recipients with HBV-related ESLD. However, no significant association between the two SNPs or haplotypes and HBV recurrence after LT was found in our research. To date, studies on the relationships between the IL-18 promoter polymorphisms ( 137G/C and 607C/A) and disease progression including HBV-related diseases are still remains controversial. Kretowski et al. reported an association of the IL-18 137 G > C but not the 607 C > A polymorphism with susceptibility to type I diabetes in a Polish population [22]. However, another study conducted in Japan revealed that patients with type I diabetes had a significantly increased frequency of IL-18 607 C/A genotype compared with control subjects, but no significant difference in the IL-18 137 allele frequencies [13]. In HBV-infected patients, the 137 G and 607 C polymorphisms increase the susceptibility to chronic progressive liver disease in Japan [23]. Patients with 137 C and 607 A may be closely linked to inhibit HBV-DNA replication in Chinese Han population [24]. However, a study from Thailand showed that genotype AA in 607 increases the risk of chronic HBV infection [25]. These contradictory results can be explained by ethnic and racial differences, association with other genetic markers, and the fact that HBV-triggered cytokine network determines the outcome of HBV infection and recurrence after LT. Accordingly, further studies in other populations are required to validate our findings. Moreover, prophylaxis against HBV recurrence post-transplantation using lamivudine and low-dose HBIG may cause the variation in the results on the association between IL-18 polymorphisms and incidence of HBV recurrence after LT. Meanwhile, it should be noted that only two SNPs in the IL-18 promoter region were investigated in our study; other SNPs in the exon, intron, 3'UTR region of IL-18 gene need to be examined in the future. Furthermore, the samples of this study was not large enough, especially, the number of patients with HBV recurrence was only 12. The real roles of IL-18 in HBV recurrence after LT in patients with HBV-related ESLD should be further investigated by large population-based studies. In summary, we did not find a significant association between the IL-18 promoter polymorphisms ( 137G/C and 607C/A) or haplotypes and HBV recurrence after LT among our study population. Other factors need to be investigated for their role in explaining and predicting HBV recurrence after LT.

## References

[1] Dienstag JL (2008). Hepatitis B virus infection. N Engl J Med.

[2] Francoz C, Belghiti J, Durand F (2007). Indications of liver transplantation in patients with complications of cirrhosis. Best Pract Res Clin Gastroenterol.

[3] Wu LM, Xu X, Zheng SS (2005). Hepatitis B virus reinfection after liver transplantation: related risk factors and perspective. Hepatobiliary Pancreat Dis Int.

[4] Jiang Z, Feng X, Zhang W, Gao F, Ling Q, Zhou L, Xie H, Chen Q, Zheng S (2007). Recipient cytotoxic T lymphocyte antigen-4 +49 G/G genotype is associated with reduced incidence of hepatitis B virus recurrence after liver transplantation among Chinese patients. Liver Int.

[5] Zhou L, Wei B, Xing C, Xie H, Yu X, Wu L, Zheng S (2011). Polymorphism in 3'-untranslated region of toll-like receptor 4 gene is associated with protection from hepatitis B virus recurrence after liver transplantation. Transpl Infect Dis.

[6] Wang WL, Zhang GL, Wu LH, Yao MY, Jin J, Jia CK, Xie HY, Zhou L, Jiang ZJ, Zheng SS (2007). Efficacy of hepatitis B immunoglobulin in relation to the gene polymorphisms of human leukocyte Fcgamma receptor III (CD16) in Chinese liver transplant patients. Chin Med J (Engl)..

[7] Mas VR, Fisher RA, Maluf DG, Archer K, Contos MJ, Mills SA, Shiffman ML, Wilkinson DS, Oliveros L, Garrett CT, Ferreira-Gonzalez A (2004). Polymorphisms in cytokines and growth factor genes and their association with acute rejection and recurrence of hepatitis C virus disease in liver transplantation. Clinical Genetics.

[8] McInnes IB, Gracie JA, Leung BP, Wei XQ, Liew FY (2000). Interleukin 18: a pleiotropic participant in chronic inflammation. Immunol Today.

[9] Okamura H, Tsutsui H, Kashiwamura S, Yoshimoto T, Nakanishi K (1998). Interleukin-18: a novel cytokine that augments both innate and acquired immunity. Adv Immunol.

[10] McGuinness PH, Painter D, Davies S, McCaughan GW (2000). Increases in intrahepatic CD68 positive cells, MAC387 positive cells, and proinflammatory cytokines (particularly interleukin 18) in chronic hepatitis C infection. Gut.

[11] Van Der Sluijs KF, Van Elden LJ, Arens R, Nijhuis M, Schuurman R, Florquin S, Kwakkel J, Akira S, Jansen HM, Lutter R, Van Der Polls T (2005). Enhanced viral clearance in interleukin-18 gene-deficient mice after pulmonary infection with influenza A virus. Immunology.

[12] Gracie JA, Koyama N, Murdoch J, Field M, McGarry F, Crilly A, Schobel A, Madhok R, Pons-Kühnemann J, McInnes IB, Möller B (2005). Disease association of two distinct interleukin-18 promoter polymorphisms in Caucasian rheumatoid arthritis patients. Genes Immun.

[13] Ide A, Kawasaki E, Abiru N, Sun F, Kobayashi M, Fukushima T, Takahashi R, Kuwahara H, Kita A, Oshima K, Uotani S, Yamasaki H, Yamaguchi Y, Eguchi K (2004). Association between IL-18 gene promoter polymorphisms and CTLA-4 gene 49A/G polymorphism in Japanese patients with type 1 diabetes. J Autoimmun.

[14] Segat L, Bevilacqua D, Boniotto M, Arraes LC, de Souza PR, de Lima Filho JL, Crovella S (2006). IL-18 gene promoter polymorphism is involved in HIV-1 infection in a Brazilian pediatric population. Immunogenetics.

[15] An P, Thio CL, Kirk GD, Donfield S, Goedert JJ, Winkler CA (2008). Regulatory polymorphisms in the interleukin-18 promoter are associated with hepatitis C virus clearance. J Infect Dis.

[16] Cheong JY, Cho SW, Oh B, Kimm K, Lee KM, Shin SJ, Lee JA, Park BL, Cheong HS, Shin HD, Cho BY, Kim JH (2010). Association of interleukin-18 gene polymorphisms with hepatitis B virus clearance. Dig Dis Sci.

[17] Wursthorn K, Wedemeyer H, Manns MP (2010). Managing HBV in patients with impaired immunity. Gut.

[18] Xie HY, Wang WL, Yao MY, Yu SF, Feng XN, Jin J, Jiang ZJ, Wu LM, Zheng SS (2008). Polymorphisms in cytokine genes and their association with acute rejection and recurrence of hepatitis B in Chinese liver transplant recipients. Arch Med Res.

[19] Giedraitis V, He B, Huang WX, Hillert J (2001). Cloning and mutation analysis of the human IL-18 promoter: a possible role of polymorphisms in expression regulation. J Neuroimmunol.

[20] Khripko OP, Sennikova NS, Lopatnikova JA, Khripko JI, Filipenko ML, Khrapov EA, Gelfgat EL, Yakushenko EV, Kozlov VA, Sennikov SV (2008). Association of single nucleotide polymorphisms in the IL-18 gene with production of IL-18 protein by mononuclear cells from healthy donors. Mediators Inflamm.

[21] Fujimori Y, Takatsuka H, Takemoto Y, Hara H, Okamura H, Nakanishi K, Kakishita E (2000). Elevated interleukin (IL)-18 levels during acute graft-versus-host disease after allogeneic bone marrow transplantation. Br J Haematol.

[22] Kretowski A, Mironczuk K, Karpinska A, Bojaryn U, Kinalski M, Puchalski Z, Kinalska I (2002). Interleukin-18 promoter polymorphisms in type 1 diabetes. Diabetes.

[23] Migita K, Sawakami-Kobayashi K, Maeda Y, Nakao K, Kondoh S, Sugiura M, Kawasumi R, Segawa O, Tajima H, Machida M, Nakamura M, Yano K, Abiru S, Kawasaki E, Yatsuhashi H, Eguchi K, Ishibashi H (2009). Interleukin-18 promoter polymorphisms and the disease progression of Hepatitis B virus-related liver disease. Transl Res.

[24] Zhang PA, Wu JM, Li Y, Yang XS (2005). Association of polymorphisms of interleukin-18 gene promoter region with chronic hepatitis B in Chinese Han population. World J Gastroenterol.

[25] Hirankarn N, Manonom C, Tangkijvanich P, Poovorawan Y (2007). Association of interleukin-18 gene polymorphism (-607A/A genotype) with susceptibility to chronic hepatitis B virus infection. Tissue Antigens.

